# Test-retest reliability of high spatial resolution diffusion tensor and diffusion kurtosis imaging

**DOI:** 10.1038/s41598-017-11747-3

**Published:** 2017-09-11

**Authors:** Pashtun Shahim, Laurena Holleran, Joong H. Kim, David L. Brody

**Affiliations:** 1 Department of Neurology, Washington University School of Medicine, St. Louis, Missouri USA; 2 Institute of Neuroscience and Physiology, Department of Psychiatry and Neurochemistry, the Sahlgrenska Academy at University of Gothenburg, Mölndal, Sweden; 3 Clinical Neurochemistry Laboratory, Sahlgrenska University Hospital, Mölndal, Sweden; 4 Hope Center for Neurological Disorders, Washington University School of Medicine, St. Louis, Missouri USA

## Abstract

We assessed the test-retest reliability of high spatial resolution diffusion tensor imaging (DTI) and diffusion kurtosis imaging (DKI). Diffusion MRI was acquired using a Siemens 3 Tesla Prisma scanner with 80 mT/m gradients and a 32-channel head coil from each of 3 concussive traumatic brain injury (cTBI) patients and 4 controls twice 0 to 24 days apart. Coefficients of variation (CoV) for DTI parameters were calculated in each DTI Studio parcellated white matter tract at 1.25 mm and 1.75 mm isotropic voxel resolution, as well as DKI parameters at 1.75 mm isotropic. Overall, fractional anisotropy had the best reliability, with mean CoV at 5% for 1.25 mm and 3.5% for 1.75 mm isotropic voxels. Mean CoV for the other DTI metrics were <7.0% for both 1.25 and 1.75 mm isotropic voxels. The mean CoV was ≤4.5% across the DKI metrics. In the commonly injured orbitofrontal and temporal pole regions CoV was <3.5% for all parameters. Thus, with appropriate processing, high spatial resolution advanced diffusion MRI has good to excellent test-retest reproducibility in both human cTBI patients and controls. However, further technical improvements will be needed to reliably discern the most subtle diffusion abnormalities, especially at high spatial resolution.

## Introduction

Diffusion tensor imaging (DTI) has proven to be an elegant noninvasive technique for assessing the microstructural characteristics of brain white matter tracts^1^. Since the introduction of DTI in 1994^2^, the technique has evolved tremendously, including faster image acquisition time, higher angular resolution, and higher spatial resolution. DTI allows the characterization of water diffusion in white mater through metrics such as fractional anisotropy (FA), axial diffusivity (AD), radial diffusivity (RD), and mean diffusivity (MD). These metrics have been shown in numerous studies to delineate subtle white matter pathologies in conditions including demyelinating diseases, movement disorders, neurodegenerative diseases, and traumatic brain injury (TBI)^3–9^.

Considerable attention has been given to the potential utility of DTI in the field of concussive TBI (cTBI), where currently there are no validated diagnostic and prognostic biomarkers. Although cTBI is considered a mild TBI, in a subset of cases postconcussive symptoms such as anxiety, attention deficit, depression, headache, and sleep disturbance may persist for months to years, especially in individuals who have had repetitive cTBIs^10–15^. cTBI is believed to primarily effect the white matter axons, which are vulnerable to the rotational acceleration forces the head is exposed to following cTBI^16–19^. Consistently, numerous studies have shown reduced FA in individuals with cTBI consistent with disruption of white matter tracts, even when conventional imaging is unremarkable^4, 11, 17, 20, 21^.

DTI is based on the assumption of Gaussian distribution of water diffusion. In biological systems, however, the complex intracellular and extracellular *in vivo* environment causes water diffusion to considerably deviate from the Gaussian pattern. Diffusion kurtosis imaging (DKI) has been a recent attempt to account for this non-Gaussian distribution in neuronal tissues^22–24^. DKI may reveal additional hallmarks of TBI^6, 25^, including astrogliosis and altered grey matter microstructure, not apparent on DTI^26^.

In order for DTI and DKI to be integrated into clinical trials of cTBI or used for individual management, the precision of the metrics needs to be established. Previous studies assessing test-retest reliability of DTI metrics have been few or limited to a small number of regions of interest, instead of whole brain white matter^27–32^. A recent study assessed the test-retest reliability of the DTI metrics at 2 mm isotropic spatial resolution in humans acquired from several different 3 Tesla scanners including 8–20 channel head coils and showed good reliability (CoV of <5%) across the entire brain white matter, but less consistency in smaller and more inferior white matter tracts, reaching CoVs of up to 10%^33^.

Recent technical developments involving stronger diffusion sensitizing gradients and more complex multichannel head coils now allow the possibility of acquiring diffusion imaging studies at higher spatial resolution, up to 1.25 mm isotropic voxel size^34^. A recent study has reported spatial resolutions up to 1.0 mm isotropic voxel size using a 7T scanner with 32-channel head coil; however, the test-retest reliability is unknown^35^. While higher spatial resolution may allow better quantification of the commonly vulnerable white matter tracts in cTBI patients, especially the inferior brain tracts such as the orbitofrontal cortex (OF) and temporal poles (TP)^4^, it may result in decreased reliability if the signal to noise ratio (SNR) is low^36, 37^. Therefore, assessing the test-retest reliability of these high spatial resolution methods is crucial, especially considering the differences in DTI metrics that have been reported in cTBI patients have been very small, ranging between 3–10%^4, 20^.

The aim of this study was therefore to assess the test-retest reliability of the DTI and DKI metrics in humans with cTBI and controls using high spatial resolution imaging protocols. We hypothesized that the reliability may be lower for cTBI patients compared to controls due to more movement in the scanner and therefore implemented approaches to reduce the influence of motion. In addition, we explicitly analyzed test-retest reliability in inferior brain regions as these regions have been shown to be highly vulnerable to image artifacts due to susceptibility^38^.

## Results

### Demographic characteristics

The demographic characteristics of the study participants are summarized in Table S1. The age of the cTBI patients (median, 25 years) and controls (median, 24 years) were essentially the same. Pairs of scans were acquired 0–24 days apart. There was no association between the CoVs and the number of days apart the pair of the scans was acquired (Fig. S3). Also, there was no association between the CoVs and difference in times of day the scans were acquired (Fig. S4).

### Test-retest reliability of 1.25 mm isotropic voxel size DTI

**Figure 1:**
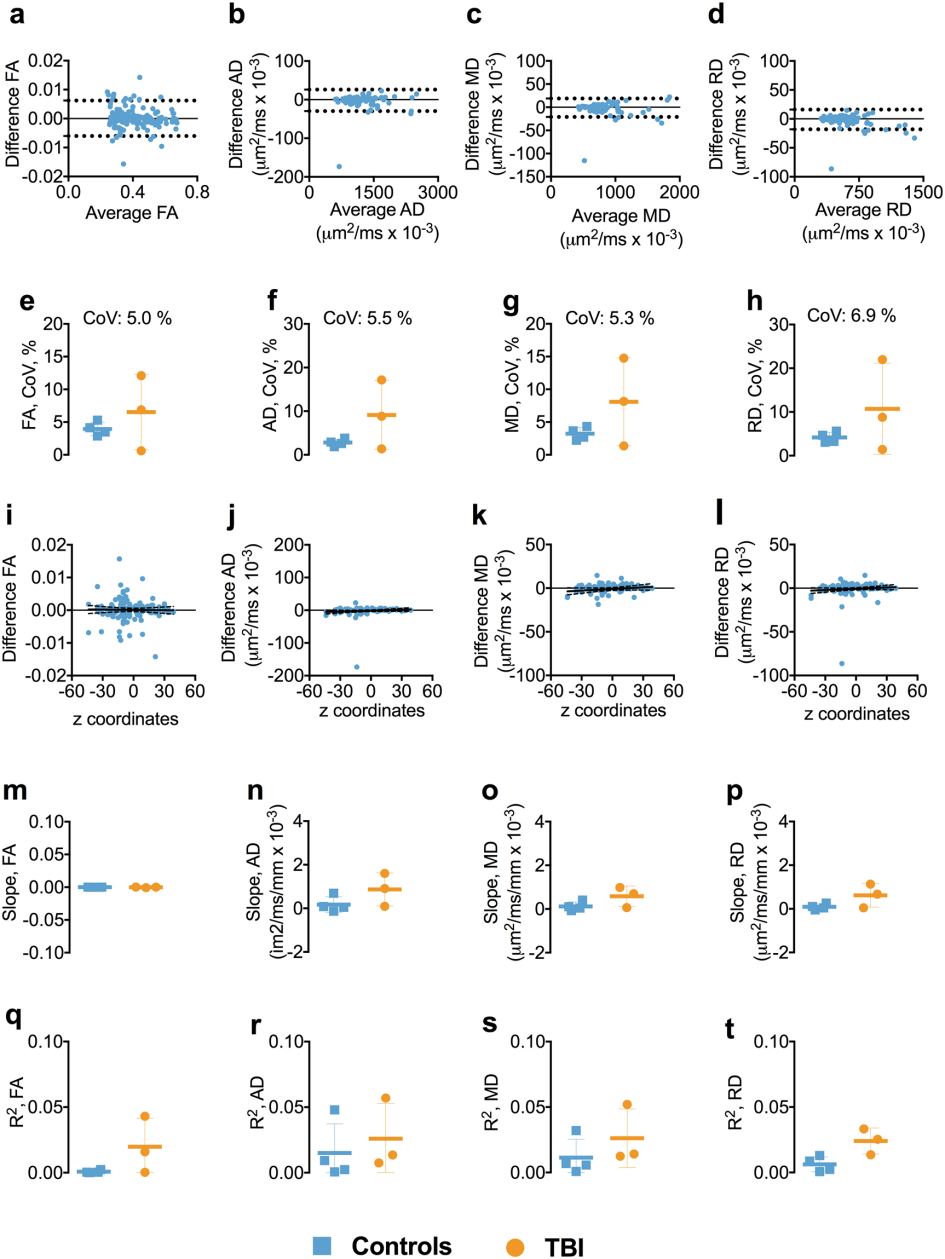
Test-retest reliability of 1.25 mm isotropic voxel size DTI. Panels a–d show the Bland-Altman plots for FA, AD, MD, and RD from one of the participants including the 95% confidence interval for the agreement between scan repeat scan. Each symbol represents a white matter region of interest from the DTI Studio parcellation. Panels e–h show the difference in coefficient of variations (CoVs) for FA, AD, RD, and MD between controls and TBI as well as the overall mean CoV for each diffusion tensor metric. Panels i–l show the linear relationships including the 95% confidence interval band between the FA, AD, MD, and RD and atlas-based white matter tract z-axis centers for one of the participants. Panels m–p show the slopes of the relationship between z-axis centers and test-retest differences. Panels q–t show the R^2^s including standard deviation for the linear relationships between the FA, AD, RD, and MD and atlas-based white matter tracts z-axis centers for controls and TBI separately. Abbreviations: FA, fractional anisotropy; AD, axial diffusivity; MD, mean diffusivity; RD, radial diffusivity; TBI; traumatic brain injury.

Test-retest reliability at 1.25 mm isotropic voxel size was generally very good. The BA plots for FA, AD, RD, and MD showed no apparent relationship between the mean of each DTI parameter and the difference between scans, indicating no systemic biases (Figs 1a–d and S5). The overall mean CoVs for FA, AD, RD, and MD, averaged over every brain white matter region of interest, were low in controls and cTBI patients, ≤4.2% and ≤9.1%, respectively (Fig. 1e–h). We noted that FA had lower CoVs than AD, RD, and MD. Furthermore, the CoVs in the controls were very consistent from subject to subject, whereas the CoVs were variable among TBI patients.

There was no overall relationship between the FA, AD, RD, and MD test-retest reliability and the localization of white matter ROIs in z-axis, despite a few outliers (Fig. 1i–l). The slopes and R^2^s for the relationships between test-retest reliability and z-axis centers for each ROI were close to zero (Fig. 1m–t). This provided initial evidence that reliability was not dramatically different in inferior brain white matter regions versus superior brain regions.

### Test-retest reliability of 1.25 mm isotropic voxel size DTI in inferior brain white matter tracts

**Figure 2:**
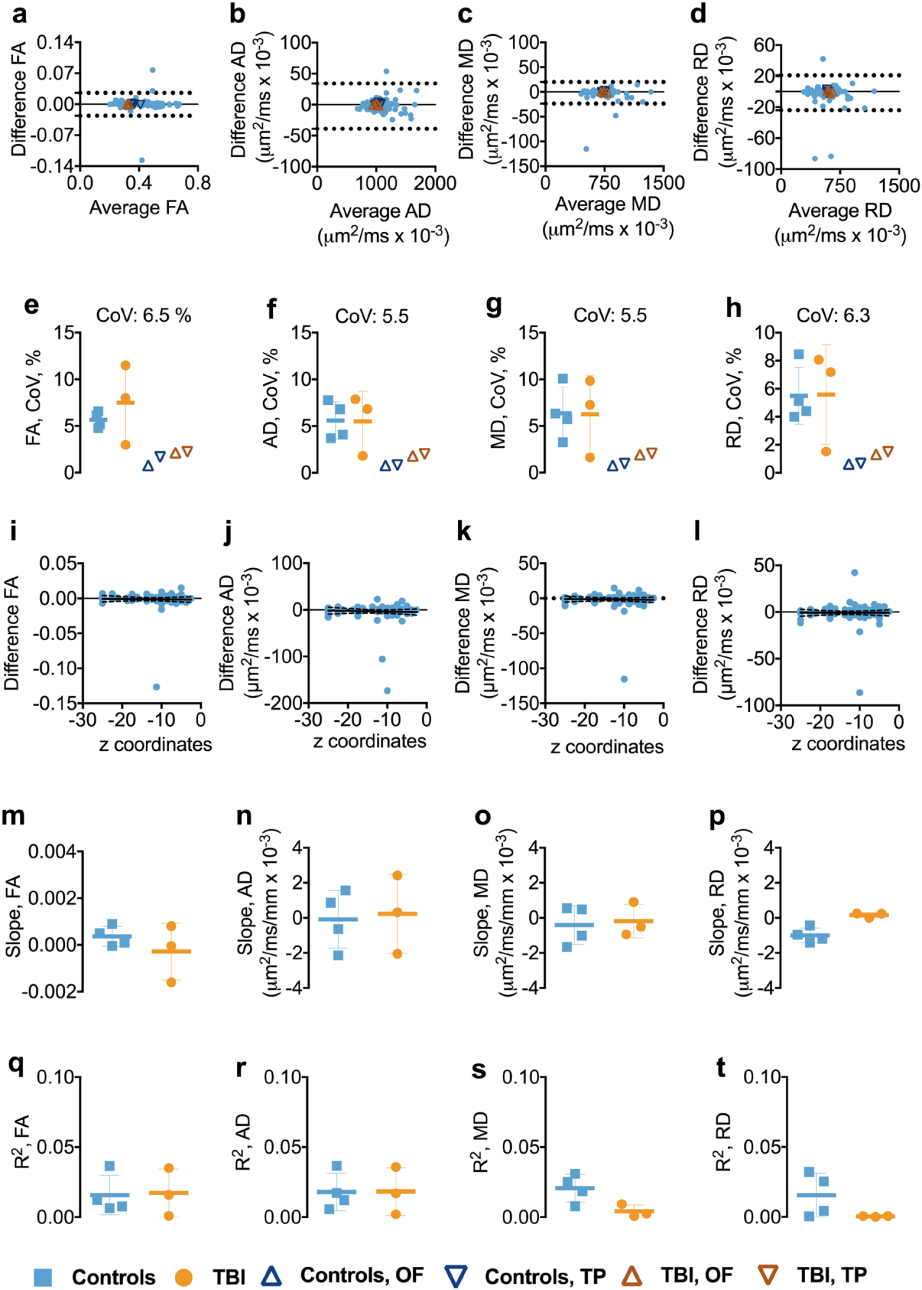
Test-retest reliability of 1.25 mm isotropic voxel size DTI in inferior brain white matter tracts. Panels a–d show the Bland-Altman (BA) plots for FA, AD, RD, and MD from one of the participants including the 95% confidence interval for the agreement between scan and repeat scan. Each symbol represents a white matter region of interest from the DTI Studio parcellation. The orange triangles in the BA plot show the agreement between scans in the OF and TP. The OF included the left and right lateral fronto-orbital gryus [LFOG] and middle fronto-orbital gyrus [MFOG] DTI Studio parcellation tracts, while the TP included the left and right superior temporal gyrus (STG), middle temporal gyrus [MTG], and inferior temporal gyrus [ITG]. Panels e–h show the difference in coefficient of variations (CoVs) for FA, AD, MD, and RD between controls and TBI as well as the overall mean CoV for each diffusion tensor metric and the mean CoVs for TP and OF. Panels i–l show the linear relationships including the 95% confidence interval band between the FA, AD, MD, and RD and atlas-based white matter tract z-axis centers for one of the participants. Panels m–p show the slopes of the relationship between z-axis centers and test-retest differences. Panels q–t show the R^2^s including standard deviation for the linear relationships between the FA, AD, MD, and RD and atlas-based white matter tract z-axis centers for controls and TBI separately. Abbreviations: FA, fractional anisotropy; AD, axial diffusivity; MD, mean diffusivity; RD, radial diffusivity; TBI; traumatic brain injury; OF, orbitofrontal; TP, temporal pole.

We separately analyzed inferior ROIs, defined as those with z axis centers <−5 mm in Talairach coordinates. In the inferior ROIs, the test-retest reliability was slightly worse than that of the whole brain (Figs 2a–d and S6). Coefficients of variation were typically in the 6% range (Fig. 2e–h). There were no substantial differences in mean CoVs for FA, AD, RD, and MD between the controls and cTBI subjects (Fig. 2e–h). The CoVs for all the DTI metrics across the groups were ≤6.5%. Overall, there was no relationship between the DTI metrics and the z-axis position of the inferior ROIs as both slopes and R^2^s for the relationship were close to zero (Fig. 2m–t).

### Test-retest reliability of DTI parameters at 1.25 mm isotropic voxel size in orbitofrontal and temporal pole white matter regions

The test-retest reliability was excellent in the commonly injured OF and TP regions; CoVs ranged from 0.6–2.2%, with controls displaying the lowest CoV, 0.6% (Figs 2e-h and S6).

### Test-retest reliability of 1.75 mm isotropic voxel size DTI

**Figure 3:**
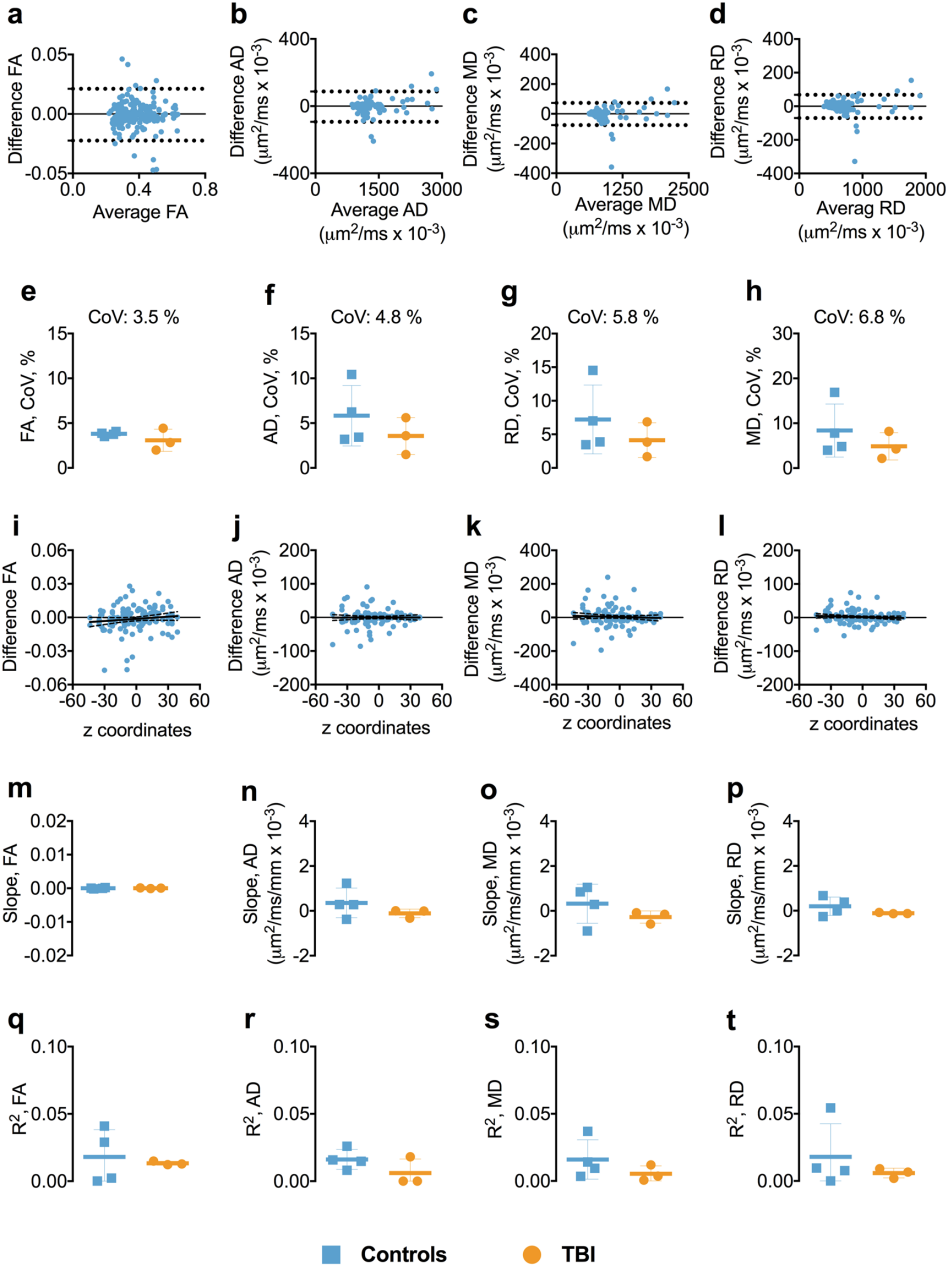
Test-retest reliability of 1.75 mm isotropic voxel. Panels a–d show the Bland-Altman plots for FA, AD, RD, and MD from one of the participants including the 95% confidence interval for the agreement between scan-rescan. Each symbol represents a white matter region of interest from the DTI Studio parcellation. Panels e–h show the difference in coefficient of variations (CoVs) for FA, AD, MD, and RD between controls and TBI as well as the overall mean CoVs for each diffusion tensor metric. Panels i–l show the linear relationships including the 95% confidence interval band between the FA, AD, MD, and RD and atlas-based white matter tracts z-axis centers for one of the participants. Panels m–p show the slopes of the relationships between z-axis centers and test-retest differences. Panels q–t show the R^2^s including standard deviation or the linear relationships between the FA, AD, MD, and RD and atlas-based white matter tracts z-axis centers for controls and TBI separately. Abbreviations: FA, fractional anisotropy; AD, axial diffusivity; MD, mean diffusivity; RD, radial diffusivity; TBI; traumatic brain injury.

Overall the test-retest reliability at 1.75 mm isotropic voxel size was very good. The BA plots showed no apparent relationship or systemic bias between the mean of each DTI parameter and at test-retest (Figs 3a–d, and S7). The mean CoVs for FA, AD, RD, and MD were below 7.0%, with FA displaying the lowest overall CoV at 3.5% (Fig. 3e–h). Also, there was no relationship between the FA, AD, RD, and MD and the anatomical localization of ROIs in the z-axis, as the slopes and R^2^s for the relationships between test-retest reliability and z-axis center position for the ROIs were either very small or close to zero (Fig. 3i–t).

### Test-retest reliability of 1.75 mm isotropic voxel size DTI in inferior brain white matter tracts

**Figure 4:**
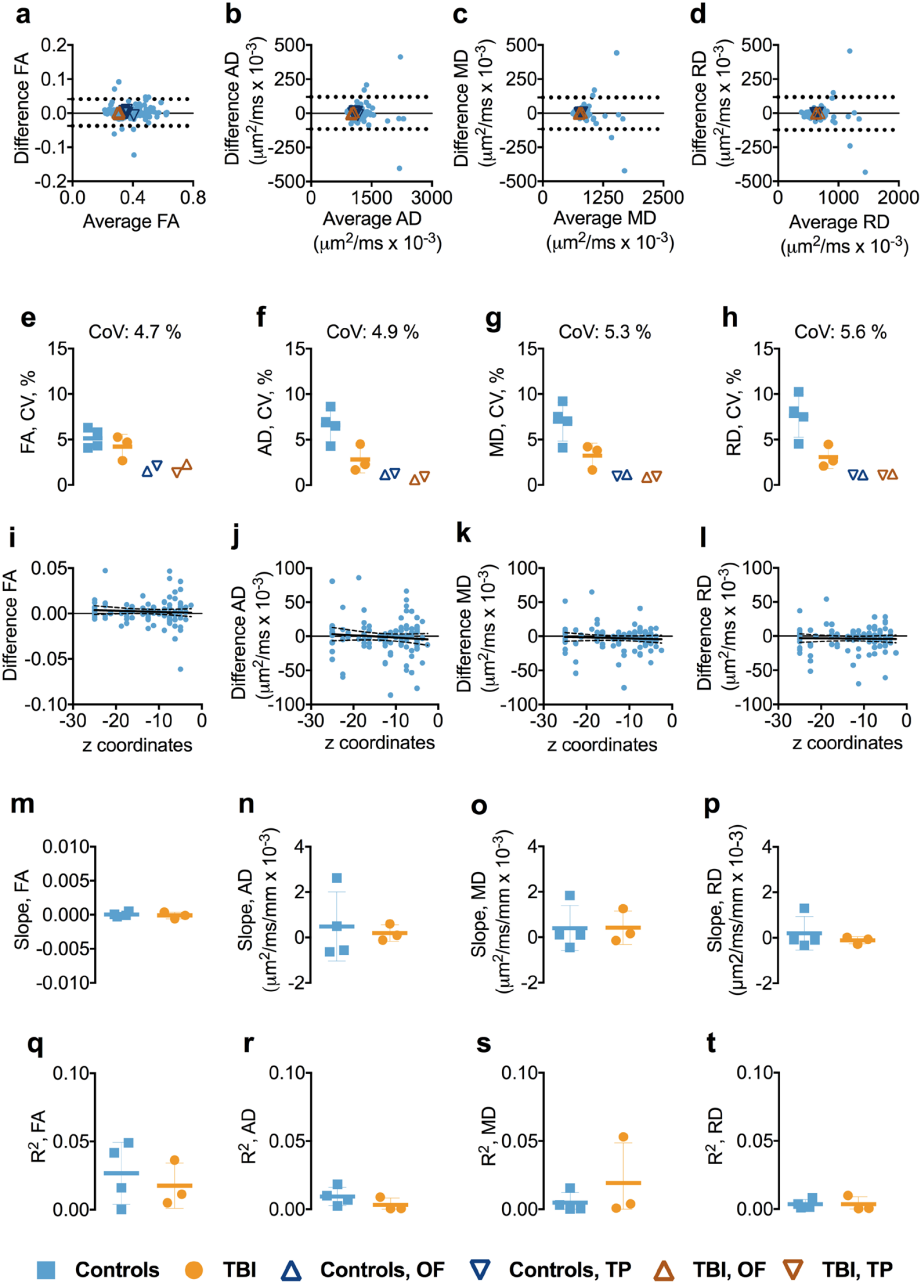
Test-retest reliability of 1.75 mm isotropic voxel in inferior brain white matter tracts. Panels a–d show the Bland-Altman (BA) plots for FA, AD, RD, and MD from one of the participants including the 95% confidence interval for the agreement between scan and repeat scan. Each symbol represents a white matter region of interest from the DTI Studio parcellation. The orange triangles in the BA plot show the agreement between scans in the OF and TP. The OF included the left and right lateral fronto-orbital gryus [LFOG] and middle fronto-orbital gyrus [MFOG] DTI Studio parcellation tracts, while the TP included the left and right superior temporal gyrus (STG), middle temporal gyrus [MTG], and inferior temporal gyrus [ITG]. Panels e–h show the difference in coefficient of variations (CoVs) for FA, AD, MD, and RD between controls and TBI as well as the overall mean CoV for each diffusion tensor metric and the mean CoVs for TP and OF. Panels i–l show the linear relationships including the 95% confidence interval band between the FA, AD, MD, and RD and atlas-based white matter tracts in z plane for one of the participants. Panels m–p show the slopes of the relationships between z-axis centers and test-retest differences. Panels q–t show the R^2^s including standard deviation for the linear relationship between the FA, AD, MD, and RD and atlas-based white matter tracts in z-axis for controls and TBI separately. Abbreviations: FA, fractional anisotropy; AD, axial diffusivity; MD, mean diffusivity; RD, radial diffusivity; TBI; traumatic brain injury; OF, orbitofrontal; TP, temporal pole.

In the inferior brain regions, the test-retest reliability was generally very good. The BA plots for FA, AD, RD, and MD showed no systemic bias (Figs 4a–d and S8). The mean CoVs for FA, AD, RD, and MD across the groups were below 5.6% (Fig. 4e–h). No relationships between the FA, AD, RD, and MD and anatomical localization of the ROIs in z-axis were observed as both slopes and R^2^s for the relationship were close to zero (Fig. 4i–t).

### Test-retest reliability of DTI parameters at 1.75 mm isotropic spatial resolution in orbitofrontal and temporal pole white matter regions

Similar to 1.25 mm isotropic voxel size DTI, the CoVs were low in the OF and TP regions, ranging from 0.6–2.2%, without apparent differences between the groups (Figs 4e–h and S8).

### Test-retest reliability of 1.75 mm isotropic voxel size diffusion kurtosis imaging

**Figure 5:**
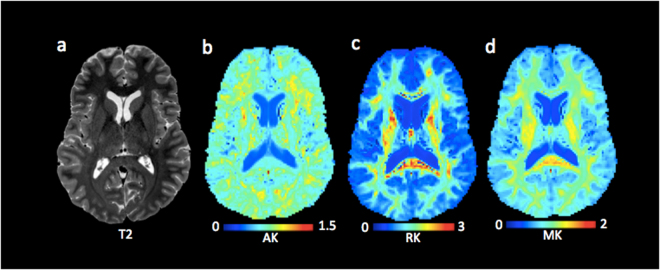
1.75 isotropic voxel size diffusion kurtosis imaging. Panels b–d show the spatial resolution of the diffusion kurtosis images. The T2-weighted image (**a**) is shown as reference. Abbreviations: AK, axial kurtosis; RK, radial kurtosis RD; MK, mean kurtosis.

Figure 5 shows an exemplar map of diffusion kurtosis parameters and a T2 weighted reference slice from one of the cTBI participants.

**Figure 6:**
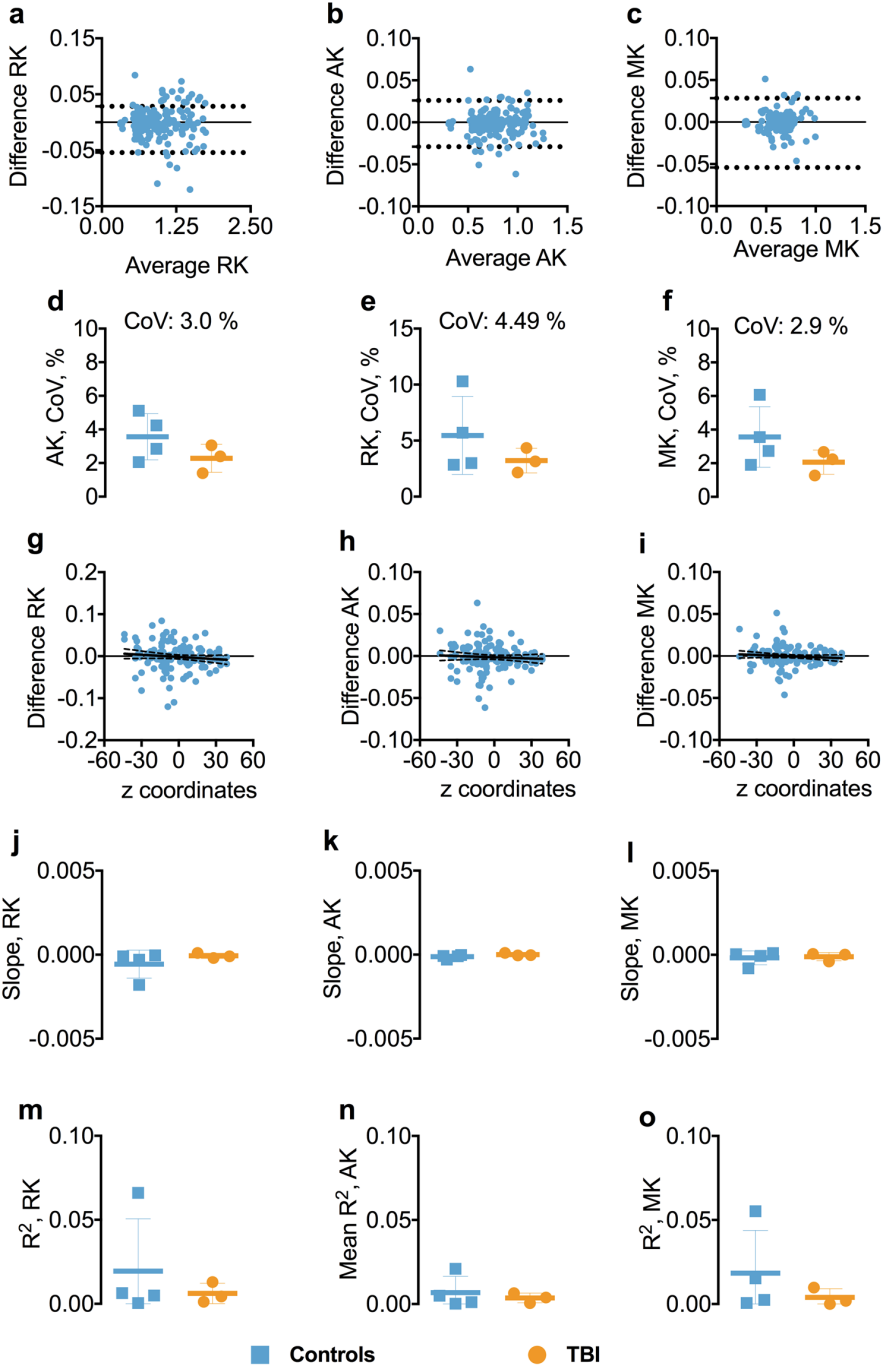
Test-retest reliability of 1.75 mm isotropic voxel diffusion kurtosis imaging. Panels a–c show the Bland-Altman plots for AK, RK, and MK from one of the participants including the 95% confidence interval for the agreement between scan 1 and scan 2. Each symbol represents a white matter region of interest from the DTI Studio parcellation. Panels d–f show the difference in coefficient of variations (CoVs) for AK, RK, and MK between controls and TBI as well as the overall mean CoV for each diffusion tensor metric. Panels g–i show the slopes of the relationship between z-axis centers and test-retest differences. Panels j–o shows the slopes and R^2^s including standard deviation for the linear relationships between the AK, MK, and RK and atlas-based white matter tracts in z-axis for controls and TBI separately. Abbreviations: TBI; traumatic brain injury; AK, axial kurtosis; RK, radial kurtosis; MK, mean kurtosis.

The BA plots for RK, AK, and MK showed robust agreement between test-retest (Figs 6a–c and S9). The mean CoVs for AK, RK, and MK were low, generally less than 4.0% (Fig. 6d–f). Also, there was no relation between the AK, MK, and RK and the anatomical localization of ROIs in z-axis as both slopes and R^2^s for the relationship were small or close to zero (Fig. 6g–o).

### Test-retest reliability of 1.75 mm isotropic voxel DKI in inferior brain white matter tracts

**Figure 7:**
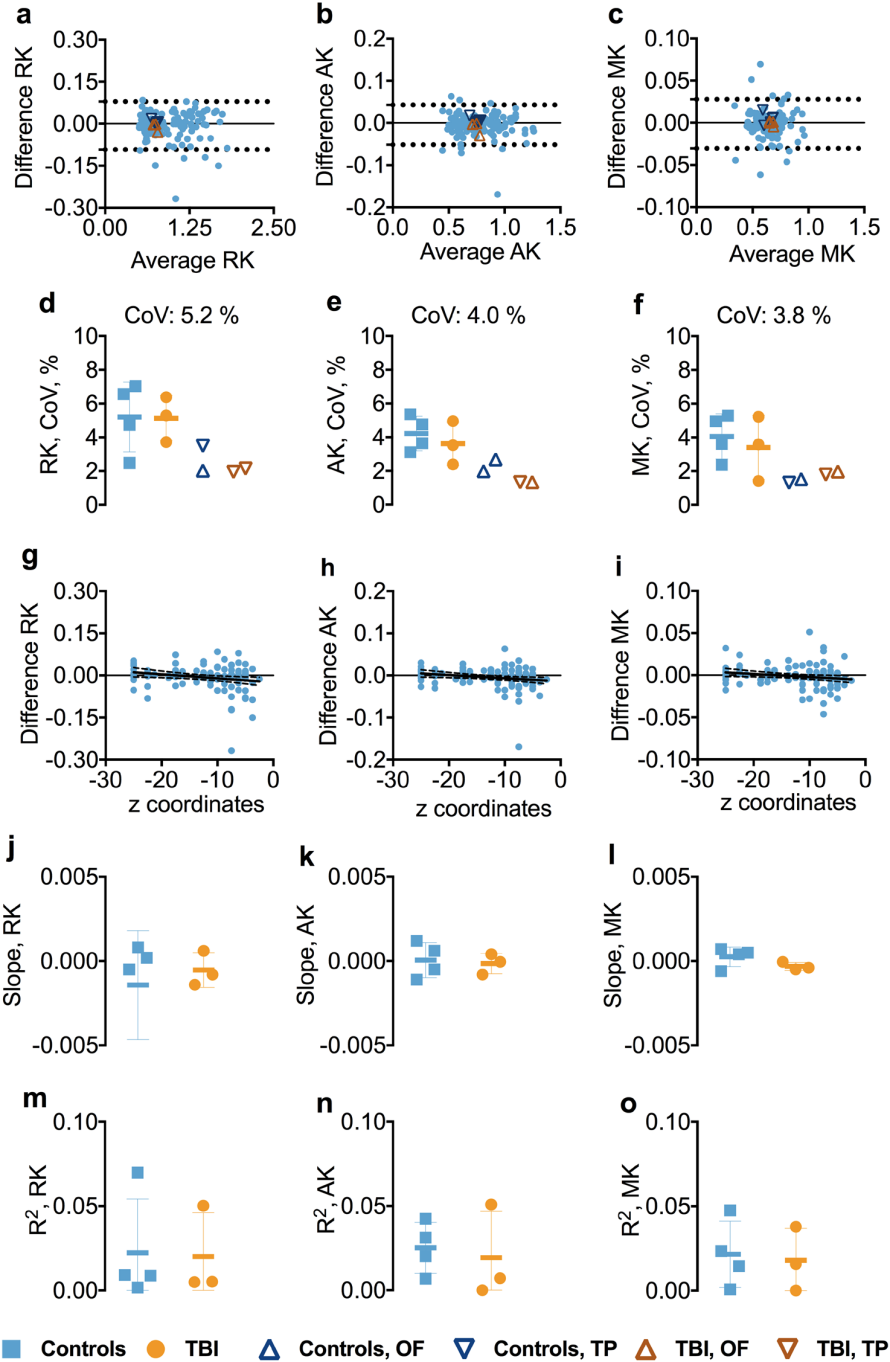
Test-retest reliability of 1.75 mm isotropic voxel diffusion kurtosis imaging in inferior brain whiter matter tracts. Panels a–c show the Bland-Altman plots for AK, RK, and MK from one of the participants including the 95% confidence interval for the agreement between scan-rescan. Each symbol represents a white matter region of interest from the DTI Studio parcellation. The orange triangles in the BA plot show the agreement between scans in the OF and TP. The OF included the left and right lateral fronto-orbital gryus [LFOG] and middle fronto-orbital gyrus [MFOG] DTI Studio parcellation tracts, while the TP included the left and right superior temporal gyrus (STG), middle temporal gyrus [MTG], and inferior temporal gyrus [ITG]. Panels d–f show the difference in coefficient of variations (CoVs) for AK, RK, and MK between controls and TBI as well as the overall mean CoV for each diffusion tensor metric and the mean CoVs for TP and OF. Panels g–i show the slopes of the relationships between z-axis centers and test-retest differences. Panels j–o show the slopes and R^2^s including standard deviation for the linear relationships between the AK, RK, and MK and atlas-based white matter tracts in z-axis for controls and TBI separately. Abbreviations: TBI, traumatic brain injury; AK, axial kurtosis; MK, mean kurtosis; RK, radial kurtosis; OF, orbitofrontal; TP, temporal pole.

The reliability of the DKI metrics remained high in the inferior ROIs. The BA plots for AK, RK, and MK indicated no systemic bias (Figs 7a–c and S10). The CoVs for all the DKI metrics were less than 5.2% (Fig. 7d–f). Overall, there was no relationship between the DKI metrics and the z-axis position of the inferior ROIs as both slopes and R^2^s for the relationships were close to zero (Fig. 7g–o).

### Test-retest reliability of DKI parameters at 1.75 mm isotropic spatial resolution in orbitofrontal and temporal pole white matter regions

CoVs for OF and TP regions were generally low across the DKI metrics, ranging between 1.3–3.5% (Figs 7d-f and S10).

## Discussion

DTI is an elegant noninvasive technique that is increasingly utilized to delineate the white matter axon architecture in the field of cTBI. However, the differences in DTI metrics between cTBI patients and controls have been very small, ranging between 3–10%^4, 20^. Thus, the ability to detect individual subject abnormalities is limited by the precision of the method. In this study, we assessed the intersession reliability of high spatial resolution DTI and DKI metrics in healthy controls and cTBI participants. The main findings of this study were: (i) the high spatial resolution diffusion imaging protocol yielded mean CoVs below 7.0% for FA, AD, RD, and MD, averaged across white matter regions, (ii) for the 1.25 mm isotropic voxel size DTI, the controls had overall lower mean CoVs than cTBI patients, (iii) Inferior ROIs, including the commonly injured temporal pole and orbitofrontal regions, had overall high reliability, (iv) DKI parameters appeared even more reliable than DTI parameters. It is important to note that in most participants there were a few outliers: ROIs with more modest test-retest reliability. Thus, interpretation of data from any single ROI in any individual subject should be made with caution.

The results of our study, with CoVs ranging from 3.5% to 7.4% for the four DTI metrics in controls and cTBI participants, generally concurs with those of prior investigations of inter-subject and inter-site DTI measurements in healthy subjects^27–32^. As expected, the cTBI patients showed higher variability across the DTI metrics, especially at the higher 1.25 mm isotropic voxel size. The effect of participant movement in the scanner may be relevant, as cTBI patients tend to display difficulty lying still due to pain, mood disorders, or attention deficits^39^. In addition, it is also possible that TBI patients’ disregulated circadian rhythms could contribute to the variability, even though we did not find an effect of time of day differences on the reliability^40, 41^.

A caveat to the many human DTI protocols has been quantifying the inferior white matter tracts such as OF and TP, as these regions are highly vulnerable to image artifact^38^. Previous studies have shown higher variability in FA in the inferior white matter tracts with CoVs up to 10% in a recent study of inter-site reliability of DTI metrics^33^. In the present study, the overall reliability of diffusion metrics was high with CoVs less than 7.0% for both spatial resolutions in the inferior brain regions, with even lower CoVs (≤2.2%) in the OF and TP. A major difference between the present study and the previous studies of reliability of advanced diffusion MRI was utilizing higher spatial resolution that may allow better quantification of inferior white matter tracts. In addition, we used dual phase encoding to compensate for susceptibility distortions that confound assessment of inferior white matter tracts such as the OF and TP, which have been reported to be vulnerable to cTBI^4^. Furthermore, optimization of brain extraction including the masking parameters may have also contributed to the overall lower CoVs, especially in the inferior ROIs.

In terms of reliability, the DKI metrics showed less variability than DTI in the present study. One plausible explanation could be that the DKI protocol includes 3 times more acquisitions than DTI, which in turn may result in better reliability. Although the constrained linear weighting method utilized herein for estimating diffusion kurtosis yielded very low CoVs, other weighted approaches such as nonlinear least square or non-Gaussian noise distribution may further improve DKI reliability^42, 43^.

The major limitation of this study was the small number of participants, especially in the cTBI group, which hindered comparison of the diffusion parameters among the groups. Due to small sample size, we also could not estimate the effect of age and gender on the reliability of the diffusion metrics. Although there was no relationship between the time of day scans were acquired and reliability, the precise relationship of circadian phase and diffusion metrics could not be established due lack of explicit measures of circadian rhythm. An additional limitation was lack of human participant motion-synchronized MR data acquisition for precise assessment of effect of motion on the diffusion metrics. Finally, we could not assess the inter-scanner variability as all scans were acquired on the same scanner.

Conclusions: Here we have shown using our proposed modified image processing protocol that advanced high spatial resolution diffusion MRI has very good but not perfect reliability in both human cTBI patients and controls. The results presented here will be useful for designing future longitudinal interventional studies including sample size estimation and interpreting individual subject-based DTI and DKI studies; resolvable differences between individual subject and a reference group or resolvable changes over time in the same individual subject are limited by the test-retest reliability of the measures. Future directions include developing gradient coils specifically for brain imaging for further enhancing the spatial resolution and improving motion correction. Also, improved multi slice radio frequency pulse sequences may reduce the scanning time and increase SNR, which would in turn result in better reliability. Thus, while test-retest reliability for diffusion imaging can be good to excellent, further technical development will be required for delineation of subtle changes relevant to concussive TBI and many other brain disorders.

## Methods

### Study participants

The study was approved by the Institutional Review Board (IRB) for human research at Washington University School of Medicine in St. Louis. All methods were performed in accordance with the relevant guidelines and regulations of the IRB at Washington University School of Medicine in St. Louis. Written informed consent was obtained from all the study participants prior to the scans.

A total of seven participants, three with cTBI and four healthy controls without a history of prior head trauma, were enrolled between 2015 and 2016 at Washington University School of Medicine in St. Louis. MRI data was acquired for each participant at 2 time points with repeat scans less than 24 days after the initial data acquisition. The demographic information is presented in Table S1. All the cTBI participants had one or more persistent postconcussive symptoms for more than three months, in other words, were scanned in the chronic stages of the injury. Thus, we do not expect changes in the state of their brain injury within the scan-rescan time frame of 24 days.

### MRI acquisition

All the MRI scans were performed on a 3 Tesla Siemens Prisma scanner, using a 32-channel radio frequency head coil. We collected T1 and T2 weighted images with 0.8 mm isotropic voxels and diffusion weighted images (DWI) with 1.25 and 1.75 mm isotropic voxels. The protocol included: scout, localizers, automatic localizer alignment, T1 (0.8 mm isotropic voxels, acquisition time 6 minutes, TR 2400 ms, TE 2.22 ms, T1 inversion time 1000 ms, flip angle 8 degrees, bandwidth 220 Hz/Px, echo spacing 7.5 ms), T2 (0.8 mm isotropic voxels, acquisition time 6 minutes, TR = 3200 ms, TE = 563 ms, flip angle = 8 degrees, bandwidth = 744 Hz/Px, echo spacing = 3.52 ms, echo train duration = 1102 ms), 1.75 mm isotropic voxel DWIs (90 non-collinear diffusion weighted gradients in Anterior-Posterior [AP] and Posterior-Anterior [PA] directions each +7 non-diffusion weighted images [7 b0s], total acquisition time of 25 minutes), and 1.25 mm isotropic voxel DWIs (30 non-collinear diffusion weighted gradients + 5 b0s, and total acquisition time of 14 minutes). A single shot 2D-DWI EPI with multiband factor 2 (https://www.cmrr.umn.edu/multiband/h) MR sequence was used to obtain diffusion data. The 1.75 mm isotropic voxel DWIs had slice thickness = 1.75 mm, TR = 7380 ms, TE = 112 ms, flip angle = 78 degree, echo spacing = 0.74 ms, bandwidth = 1488 Hz/Px, and EPI factor = 120 and total 90 directions non-collinear diffusion sensitizing gradient schemes (30 directions per b-value shell at 1000 s/mm^2^, 2000 s/mm^2^ and 3000 s/mm^2^). The 1.25 mm isotropic DWIs had slice thickness = 1.25 mm, TR = 10937 ms, TE = 118.40 ms, flip angle = 76 degrees, echo spacing = 0.74 ms, bandwidth = 744, and EPI factor = 168 and 30 diffusion sensitizing gradient schemes with b-values = 1000 s/mm^2^. The b-values and b-vectors for both 1.25 and 1.75 mm isotropic voxel sizes are attached as Appendix 1.

Diffusion data were acquired using single shot 2D SE-DWI EPI with multi-band factor 2 (https://www.cmrr.umn.edu/multiband/). The flip angle was optimized to minimize any harmful specific absorption rate or so called tissue heating. The currently employed 3D T1 MPRAGE (magnetization-prepared 180 degrees radio-frequency pulses and rapid gradient-echo) and 3D T2 SPACE (sampling perfection with application optimized contrasts using different flip angle evolution) pulse sequence are aimed for high spatial resolution, which is 0.8 mm isotropic in this study. Therefore, to avoid excessive tissue heating, the flip angle was set at 8 degrees for T1 and varied from 8 to 60 degrees for T2.

We attempted to acquire 1.25 mm isotropic voxel size DKI; however, SNRs at higher b-values were insufficient. Therefore, the DKI in the present study were acquired using 1.75 mm isotropic voxels in order to allow adequate SNR. The average SNR for 1.25 and 1.75 mm isotropic voxel size calculated for three brain images is shown in Tables S2 and S3.

### DTI processing

Once all images were collected, they were converted to NIfTI format using dcm2nii program (http://nitrc.org/projects/dcm2nii). The T1 and T2 weighted images were manually aligned to anterior and posterior commissures (AC-PC) for consistency of physical orientation using mipav (http://mipav.cit.nih.gov). In the next step, a quality check on every data set, including DKI was run to detect image slices with severe signal dropout due to head motion. If the mean signal in any slice was more than four standard deviations below the mean signal for that slice on the rest of the DWIs, the entire b-vector series was excluded (Fig. S1). The number of b-vector series excluded for each patient is reported in Table S1. The DIFF_PREP module in TORTOISE (https://science.nichd.nih.gov/confluence/display/nihpd/TORTOISE) was used to correct AP and PA data sets separately for subject motion and eddy current distortion as well as susceptibility induced EPI distortion. Further distortion correction was done in DR-BUDDI module of TORTOISE using the AP and PA data^38, 44–47^. Next, the multiple non-diffusion weighted images (b0 images) were averaged into one, and then this image was merged with DWIs to estimate diffusion metrics.

**Figure 8:**
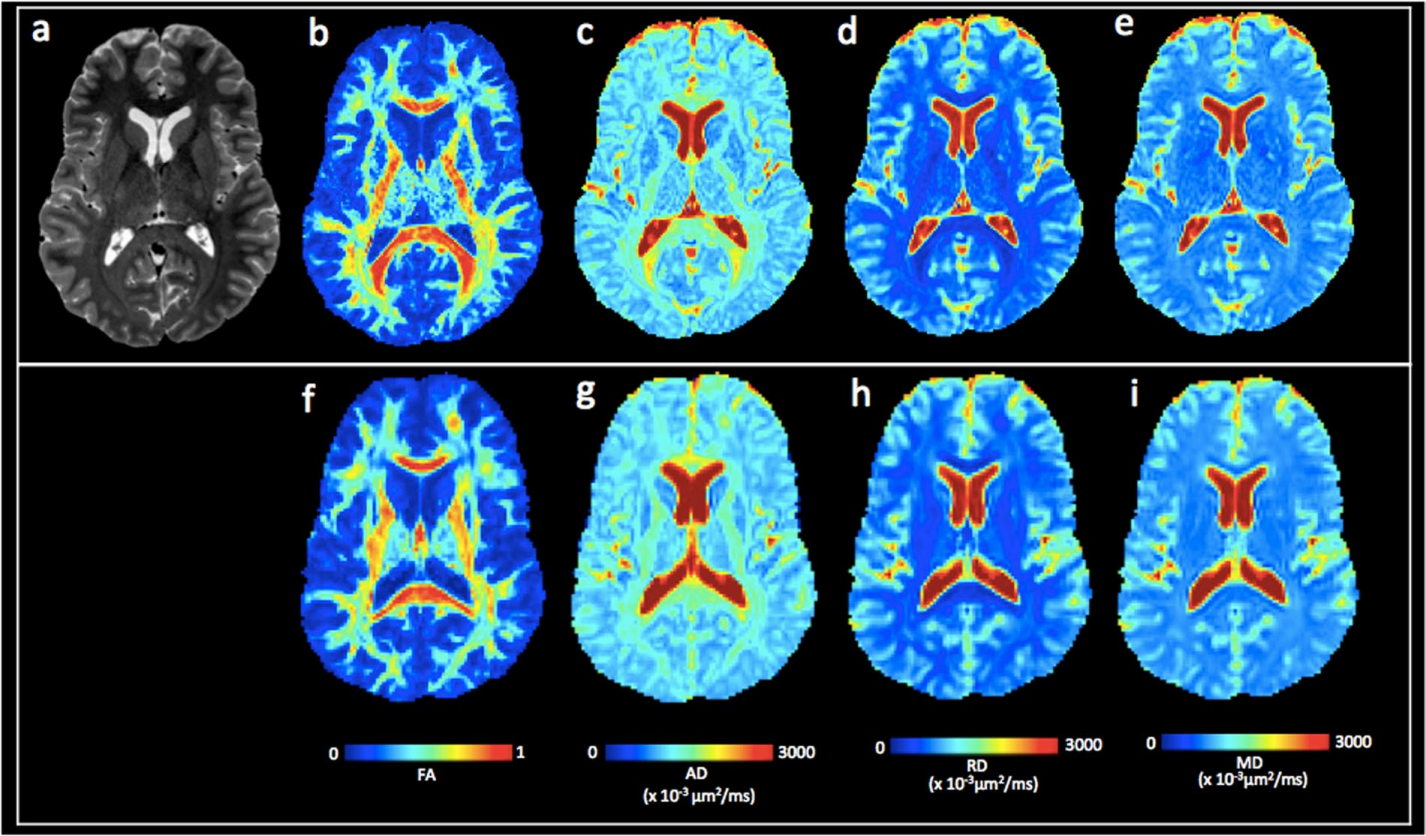
Comparison of diffusion tensor imaging at 1.25 vs. 1.75 mm isotropic voxel size. (**a**) T2 weighted image for a cTBI subject. (**b**–**e**) 1.25 mm isotropic voxel DTI parameter maps: (**b**) FA, (**c**) AD, (**d**) RD, and (**e**) MD. (**f**–**i**) 1.75 mm isotropic voxel DTI parameter maps: (**f**) FA, (**g**) AD, (**h**) RD, and (**i**) MD. Abbreviations: FA, fractional anisotropy; AD, axial diffusivity; RD, radial diffusivity; MD, mean diffusivity.

The registered images were skull-stripped using a combination of the FSL Brain Extraction Tool and the DIFF_CALC module of TORTOISE^48^. In order to remove the remaining unwanted noise or non-brain tissue voxels after the brain extraction, the brain masking parameters for both 1.25 and 1.75 mm isotropic voxels were optimized. For the 1.25 mm isotropic DWIs, the erosion factor (ef) threshold of 2, fraction intensity threshold or f bet = 0.2 (smaller values give larger brain outline estimates), Noise threshold (N) = 4, and g bet value = 0.0 (positive values give larger brain outline at bottom, smaller at top) were assessed as optimal for diminishing the non-brain voxels. For the 1.75 mm isotropic DWIs, the following masking parameters were assessed as optimal: ef = 3, f bet = 0.3, g = 0.0 and N = 4 for diminishing the non-brain voxels. The masks were further optimized in the ImageJ (http://imagej.nih.gov/ij/docs/guide) by comparing the masked images to the unmasked raw DWIs. The Otsu threshold method^49^ was applied on the unmasked raw images to separate them from their background and facilitate comparison with the masked images. The regions or areas where the FSL + DIFF_CALC masking was excessively aggressive were manually recreated from the unmasked raw DWIs, especially in the inferior regions and temporal poles. Example of the masking process is shown in Fig. S2. The optimized mask was then applied to the raw unmasked DWIs to calculate the DTI and DKI parameters in DIFF_CALC. DTI metrics were estimated in the DIFF_CALC module of TORTOISE, using a non-linear least square method^50^, and robust estimation of tensor by outlier rejection^51^. Diffusion kurtosis metrics were estimated using diffusion kurtosis estimator^23^. The currently employed diffusion kurtosis estimator utilizes constrained linear weighting method. The detailed diffusion kurtosis estimation can be found here (http://academicdepartments.musc.edu/cbi/dki/index.html). Examples of the processed DTI from one of the cTBI patients are shown in Fig. 8.

### Parcellation

The brain parcellation maps were acquired from DTI studio (http://www.mristudio.org). The DTI images were processed with the DiffeoMap module of the DTI studio using FA maps to perform large deformation diffeomorphic metric mapping (LDDMM) transformation. After the transformation, parcellation maps that were obtained from the remote server were applied to the intensity corrected FA images. A threshold of FA >0.2 was applied to exclude gray matter and voxels containing substantial partial volume effects with gray matter or CSF. Next, the FA-based brain parcellation map was applied to all diffusion metrics for quantitative analysis using the ROI editor module of the DTI studio.

### Statistics

As overall measures of precision, we calculated the CoV across all the DTI and DKI metrics for each individual participant. The CoV was obtained for each diffusion metric in the whole brain white matter as standard deviation divided by the average. Bland-Altman (BA) plots were used to examine the test-retest reliability across the DTI and DKI metrics as a function of the means of the metrics. To assess whether test-retest reliability of DTI and DKI metrics was worse in inferior brain regions as compared to more superior regions, we assessed the relationship between reliability and the z-axis position for each ROI. The *MRI Atlas of Human White Matter* ^52^ was used for determining the z coordinates for each white matter ROI (JHU_MNI_SS_BPM_TypeII_V2.1). The potential confounding effect of circadian rhythm (time of day the scans were acquired) and number of days between scan-rescan on the CoVs were examined using Spearman’s rank correlation. All statistical tests and plots were implemented in GraphPad Prism 7.0 (GraphPad Inc., San Diego, CA).

### Data availability

The datasets generated during and/or analyzed during the current study are available from the corresponding author on reasonable request.

## Electronic supplementary material

Supplementary Information
