# Supplementary material for: Test-retest reliability of high spatial resolution diffusion tensor and diffusion kurtosis imaging
Source: Sci Rep. 2017 Sep 11;7:11141. doi: 10.1038/s41598-017-11747-3 (PMC5593980; doi:10.1038/s41598-017-11747-3)
Supplement: Supplementary file 1 — Supplementary Information [file 41598_2017_11747_MOESM1_ESM.pdf]

Title: Test-retest reliability of high spatial resolution diffusion tensor and diffusion kurtosis imaging

Authors: Pashtun Shahim[1, 2,3], MD, PhD, Laurena Holleran[1], PhD, Joong H. Kim[1], PhD, David L. Brody[1, 4], MD, PhD

Affiliations:

[1]Department of Neurology, Washington University School of Medicine, St. Louis, Missouri, USA.

[2]Institute of Neuroscience and Physiology, Department of Psychiatry and Neurochemistry, the Sahlgrenska Academy at University of Gothenburg, Mölndal, Sweden.

[3]Clinical Neurochemistry Laboratory, Sahlgrenska University Hospital, Mölndal, Sweden.

[4]Hope Center for Neurological Disorders, Washington University School of Medicine, St. Louis, Missouri, USA.

Correspondence: Pashtun Shahim, MD, PhD
Washington University School of Medicine
660 S Euclid Avenue, Box 8111
St Louis, MO 63110 USA
Tel: +1 (314) 938 7552
E-mail: shahimp@wustl.edu

Supplementary Material:

Table S1. Demographic characteristic and DTI acquisition time and date of the study participants

Case	Age, years	Sex	Time of day, h		Days apart	No. of excluded volumes[a]		
			Scan 1	Scan 2		1.25 mm DTI[b]	1.75 mm DTI[c]	1.75 mm DKI[d]
Control 1	27	Female	14:39	11:14	5.0	4,4	0,0	0,0
Control 2	24	Male	15:45	15:03	2.0	0,1	0,0	0,2
Control 3	24	Female	16:51	15:37	1.0	1,1	2,0	0,3
Control 4	34	Male	14:34	16:48	16.0	0,1	0,0	2,0
TBI 1	28	Male	9:53	10:30	6.0	2,2	0,0	0,2
TBI 2	18	Female	18:22	19:22	0.0	1,0	1,0	3,5
TBI 3	24	Female	08:11	08:13	24.0	2,2	0,0	1,2

Abbreviations: TBI, traumatic brain injury.
[a]Image volumes excluded due to signal dropout at initial and second scan.
[b]125 mm isotropic voxel size diffusion tensor imaging.
[c]1.75 mm isotropic voxel size diffusion tensor imaging.
[d]1.75 mm isotropic voxel size diffusion kurtosis imaging.

Table S2. Signal to noise ratio at 1.25 mm isotropic resolution

Case	Signal to Noise Ratio											
	1.25 mm DTI, scan1						1.25 mm DTI, scan 2					
	CC		ITG-WM		SFG-WM		CC		ITG-WM		SFG-WM	
	*b*0	*b*1000	*b*0	*b*1000	*b*0	*b*1000	*b*0	*b*1000	*b*0	*b*1000	*b*0	*b*1000
Control 1	14.7	5.0	20.0	4.0	27.5	7.0	14.7	5.2	17.0	5.1	24.0	9.1
Control 2	14.8	7.7	14.0	6.7	29.9	15.0	12.6	6.9	15.0	6.7	27.0	14.3
Control 3	27.0	11.6	19.9	9.3	29.0	14.0	17.0	9.4	15.0	7.5	28.5	14.0
Control 4	15.3	8.0	11.0	5.3	26.0	14.0	19.0	9.0	39.0	6.7	24.0	13.0
TBI 1	16.4	8.8	12.7	6.4	28.0	14.7	28.0	14.7	13.0	6.3	28.0	14.9
TBI 2	18.5	10.0	22.0	10.0	40.0	20.0	17.0	9.0	20.0	9.9	33.5	16.0
TBI 3	18.0	9.1	18.0	8.6	27.0	14.0	17.6	8.3	19.0	8.5	26.5	12.6

SNR was calculated by the formula ([signal intensity/standard deviation of background noise] x 0.66). The average SNR for 5 b0s and 30 diffusion-weighted images acquired at *b*1000 s/mm^2 is shown here for three brain regions. Abbreviations: TBI, traumatic brain injury; SNR, signal to noise ratio; DTI, diffusion tensor imaging; CC, corpus callosum anterior; ITG-WM, inferior temporal gyrus white matter; SFG-WM, superior frontal gyrus white matter.

Table S3. Signal to noise ratio at 1.75 mm isotropic resolution

Case	Signal Noise Ratio																							
	1.75 mm DTI, scan1												1.75 mm DTI, scan 2											
	CC				ITG-WM				SFG-WM				CC				ITG-WM				SFG-WM			
	*b*0	*b*1	*b*2	*b*3	*b*0	*b*1	*b*2	*b*3	*b*0	*b*1	*b*2	*b*3	*b*0	*b*1	*b*2	*b*3	*b*0	*b*1	*b*2	*b*3	*b*0	*b*1	*b*2	*b*3
Control 1	43.0	21.0	16.0	12.0	70.5	29.0	16.0	9.3	66.7	35.0	22.0	17.0	41.0	22.0	16.0	16.0	82.0	38.0	20.0	12.0	89.6	44.0	28.0	22.0
Control 2	41.3	23.0	16.5	13.0	68.0	32.0	18.0	10.0	80.0	41.0	27.5	21.0	42.0	22.0	15.0	13.0	74.0	33.3	16.0	11.0	78.0	40.0	26.0	21.0
Control 3	78.0	25.7	17.0	12.0	78.0	29.0	20.0	13.0	80.0	40.0	23.0	16.0	47.1	24.0	17.0	14.0	62.0	27.5	15.0	9.5	79.0	41.0	26.0	20.0
Control 4	42.0	22.0	16.0	12.0	32.0	16.0	9.5	7.5	66.0	37.0	25.0	19.0	45.0	22.0	16.0	13.0	32.0	14.0	8.6	6.5	81.0	38.0	23.0	16.0
TBI 1	60.0	26.0	19.0	14.0	55.0	27.0	15.0	9.6	85.0	44.0	29.0	23.0	55.5	24.0	19.0	14.0	55.0	26.0	15.0	9.5	89.0	47.0	31.0	24.0
TBI 2	54.0	28.0	19.0	15.0	75.0	32.0	18.0	11.0	103.7	51.0	31.0	22.0	43.0	23.0	16.0	13.0	65.0	30.0	16.0	9.0	89.0	41.0	25.0	17.6
TBI 3	74.0	36.0	22.0	16.0	86.0	33.0	18.0	10.0	70.0	35.6	22.6	17.0	44.0	22.0	15.0	12.0	74.0	31.0	16.0	9.5	67.0	34.0	22.0	16.0

SNR was calculated by the formula (signal intensity/standard deviation of background noise x 0.66). The average SNR for 7 b0s and 90 diffusion weighted images at *b* =1000, 2000, and 3000 s/mm^2 shown here for three brain regions. Abbreviations: SNR, signal to noise ratio; DTI, diffusion tensor imaging; CC, corpus callosum anterior; ITG-WM, inferior temporal gyrus white matter; SFG-WM, superior frontal gyrus white matter; b1, b=1000 s/mm^2; b2, b=2000 s/mm^2; b3, b=3000 s/mm^2

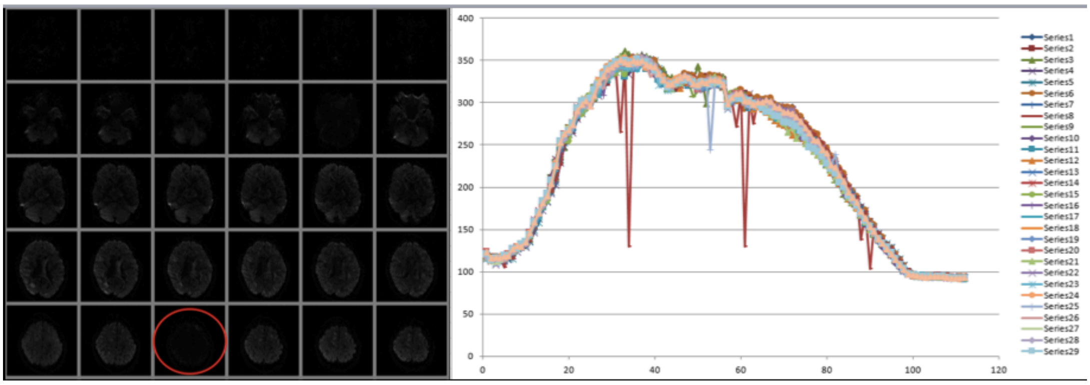

Fig. S1. Quality check for signal dropout. An example of a quality control plot for one subject's diffusion weighted imaging data at 1.75 mm isotropic voxel size with phase encoding in anterio-posterior direction at b=1000 s^2/mm. The red circle shows the signal dropout in one of the slices. Each series on the right plot indicates one diffusion-weighted scan; there are 30 series because 30 diffusion directions (b-vectors) were assessed. The y-axis shows the mean signal for the whole slice, and the x-axis indicates the slice number. Here slice numbers 32, 34, 54, 59, 61, 88, and 90 in series 8 and slice 53 in series 25 have clearly reduced signals. These two series were deleted, and the diffusion parameters were calculated from the remaining 28 diffusion-weighted scans. This type of quality check was carried out for every data set.

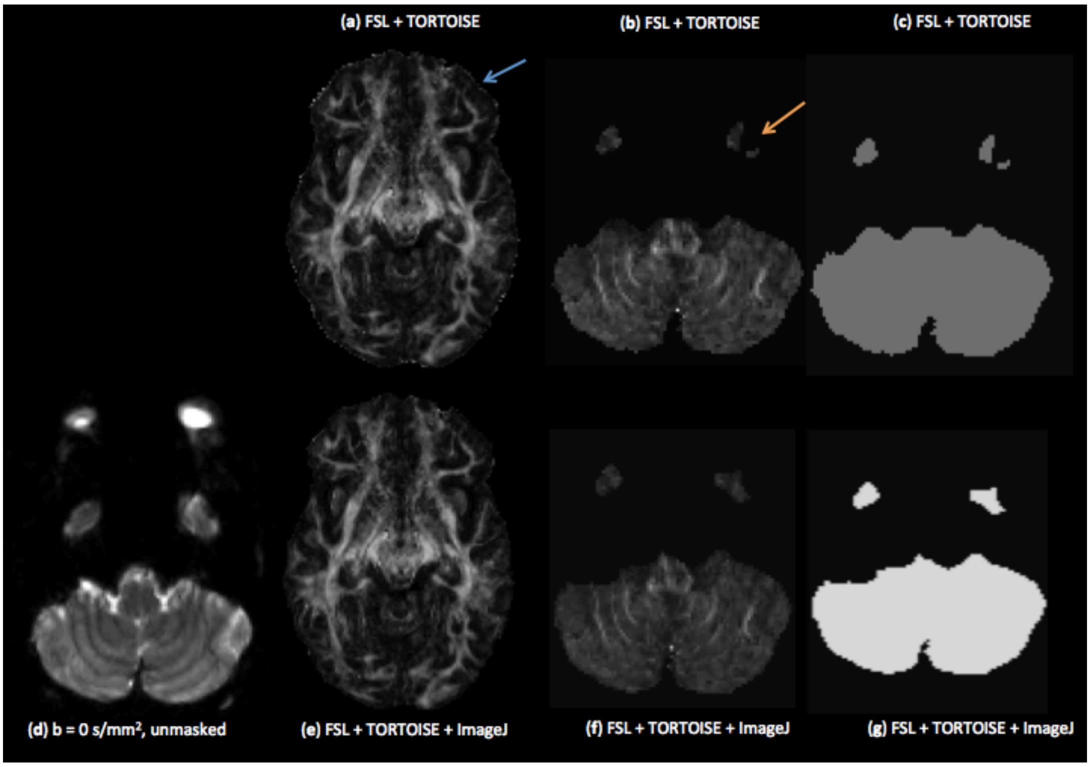

Fig. S2. Optimization of the brain extraction and masking at 1.25 mm isotropic voxel size. (**a**) Fractional anisotropy map in a slice containing orbitofrontal white matter tracts. FSL + TORTOISE masking result in occurrence of speckles or non-brain tissue (blue arrow) throughout the slices. (**b**) Excessive brain tissue erosion in the inferior brain regions (orange arrow indicating the inferior regions of temporal pole). (**c**) FSL + TORTOISE mask applied. (**d**) Unmasked b = 0 s^2/mm image for reference. (**e**) Example of the optimized masking (FSL+TORTOISE+ImageJ) in the orbitofrontal regions removing speckles. (**f**) Restored inferior brain regions including temporal pole resulting in reduced tissue erosion. (**g**) The optimized mask that was created and applied for final analyses.

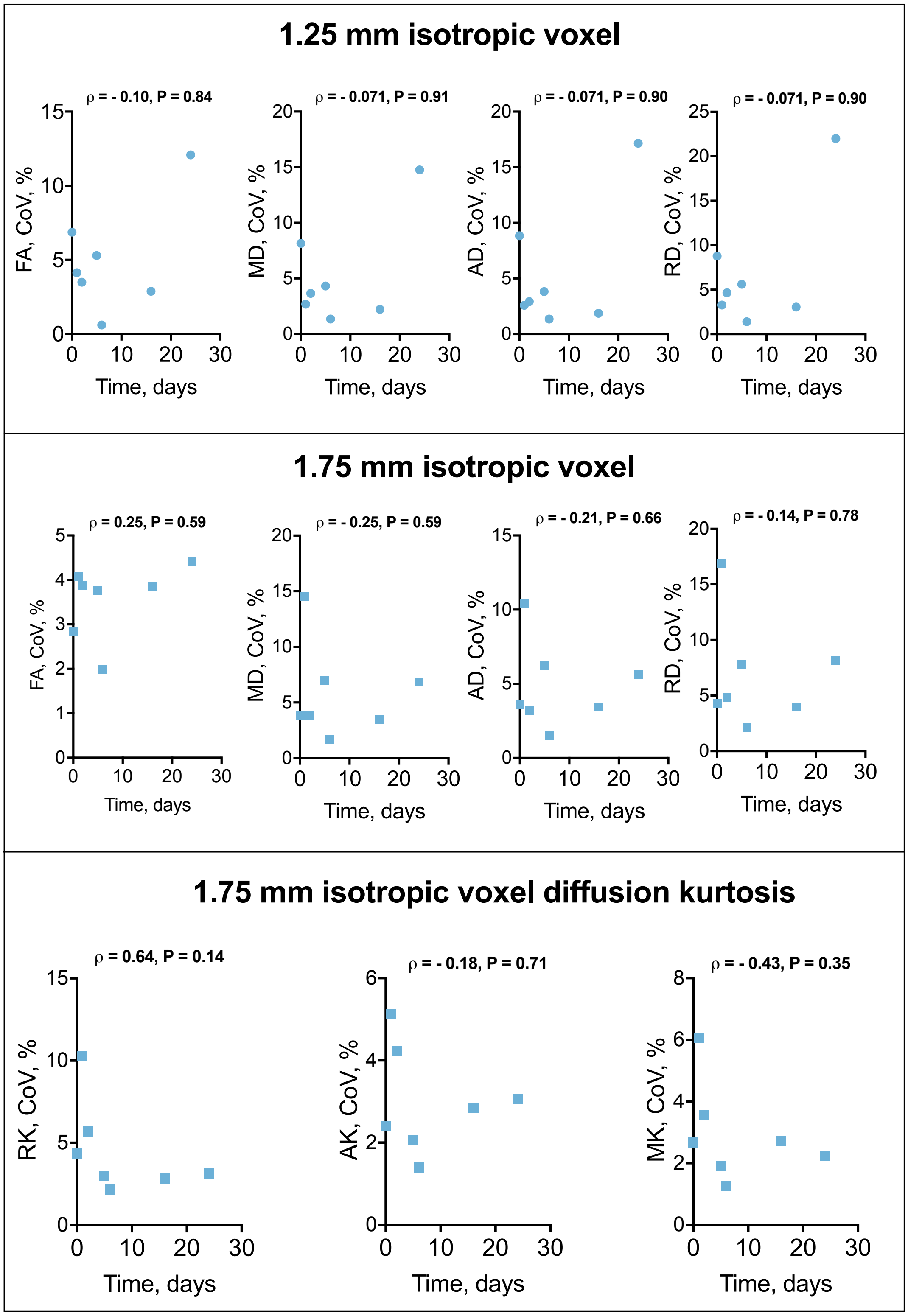

Fig. S3. Association between the coefficients of variation (CoV) and the number of days between initial scan and repeat scan. The P values are from the Spearman's rank correlations. Abbreviations: FA, fraction anisotropy; RD, radial diffusivity; AD, axial diffusivity; RK, radial kurtosis; AK, axial kurtosis; MD, mean kurtosis.

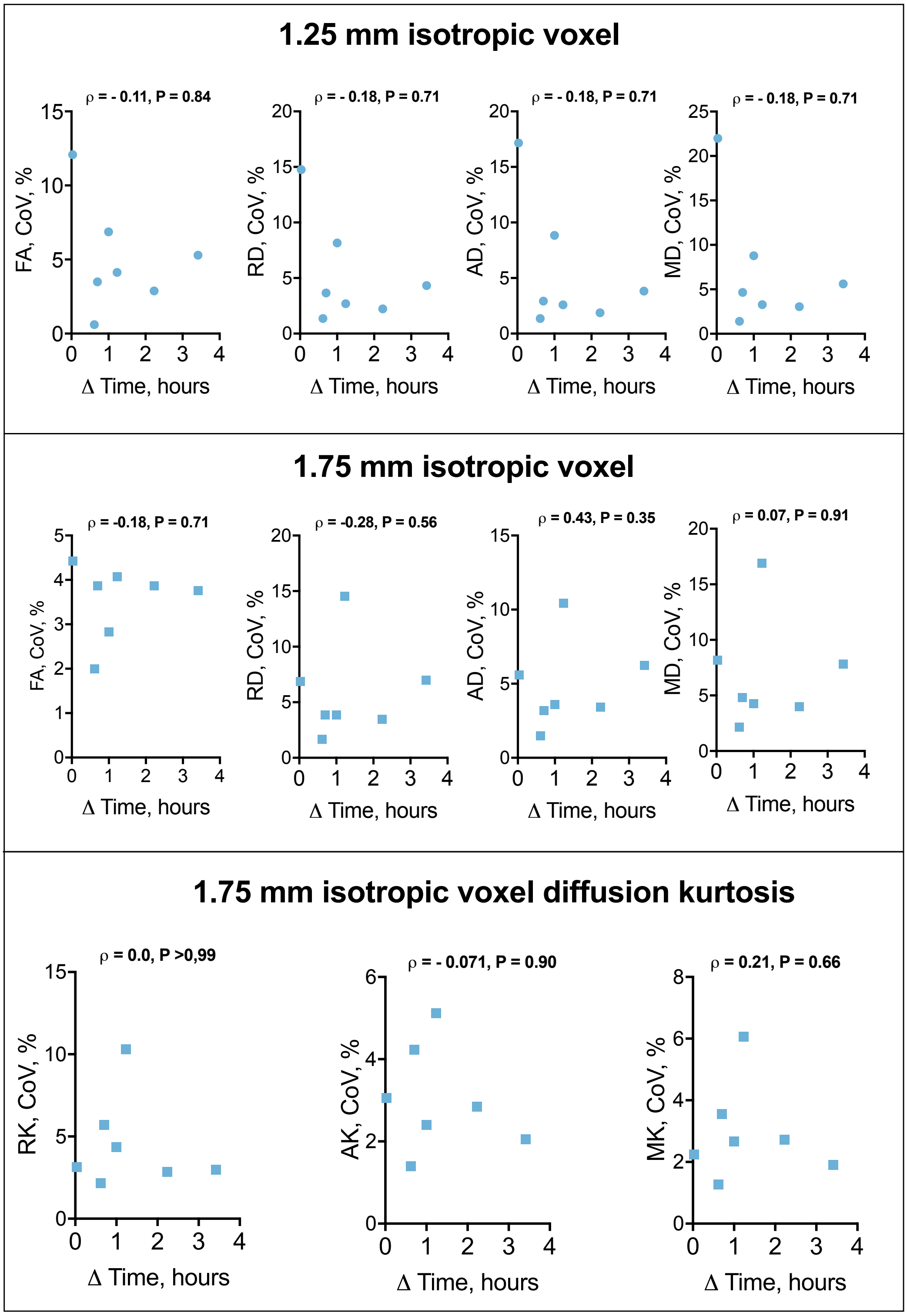

Fig. S4. Association between the coefficients of variation (CoV) and circadian time of scans. X-axes show the absolute difference in time of day (hours) between the initial scan and repeat scan. The P values are from the Spearman's rank correlations. Abbreviations: FA, fraction anisotropy; RD, radial diffusivity; AD, axial diffusivity; RK, radial kurtosis; AK, axial kurtosis; MD, mean kurtosis.

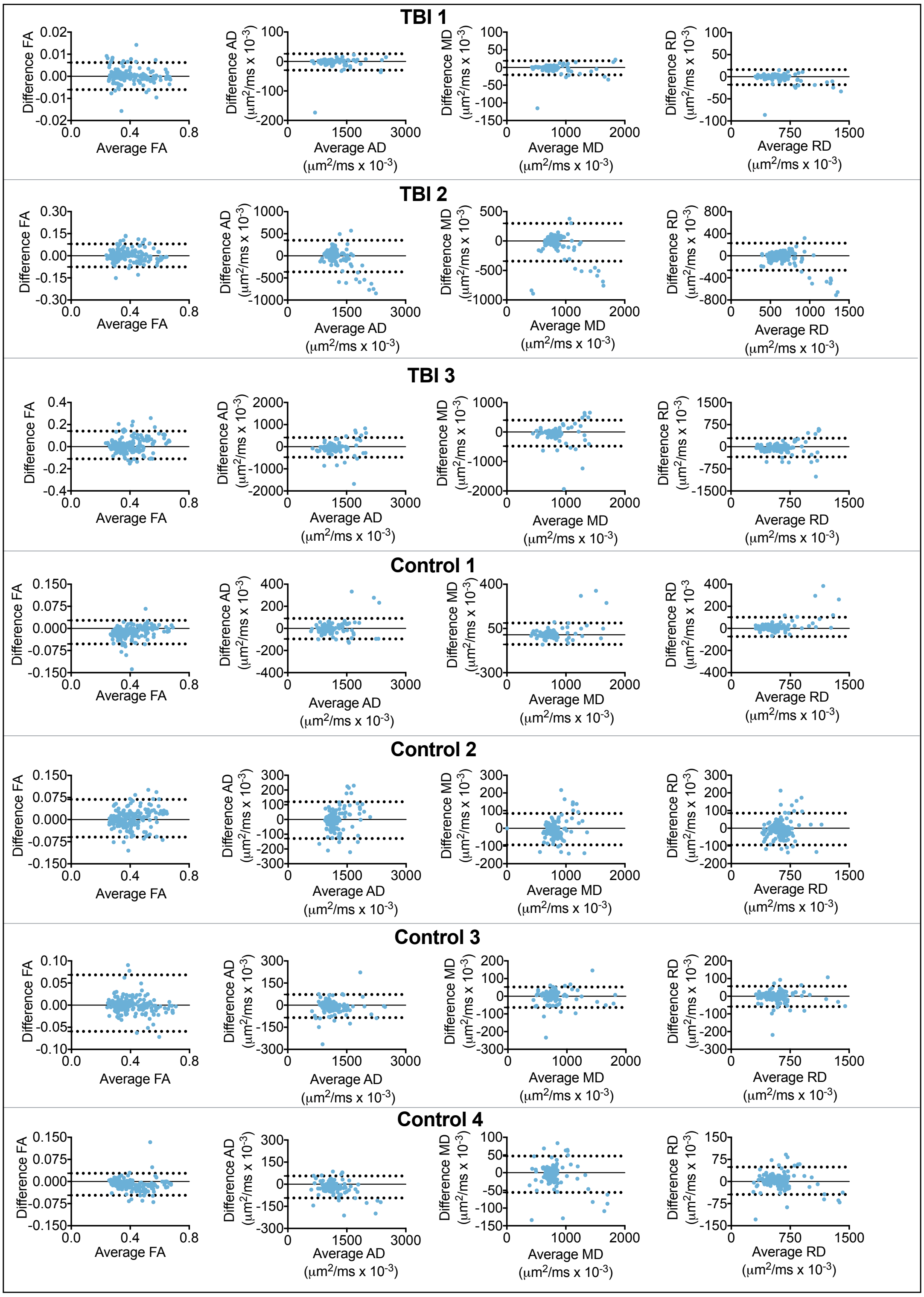

Fig. S5. Test-retest reliability of DTI with 1.25 mm isotropic voxel size in brain white matter tracts. Bland-Altman plots showing minimal systematic bias in all 7 participants. Each symbol represents a white matter region of interest from the DTI Studio parcellation. Abbreviations: FA, fractions anisotropy; AD, axial diffusivity; MD, mean diffusivity; RD, radial diffusivity.

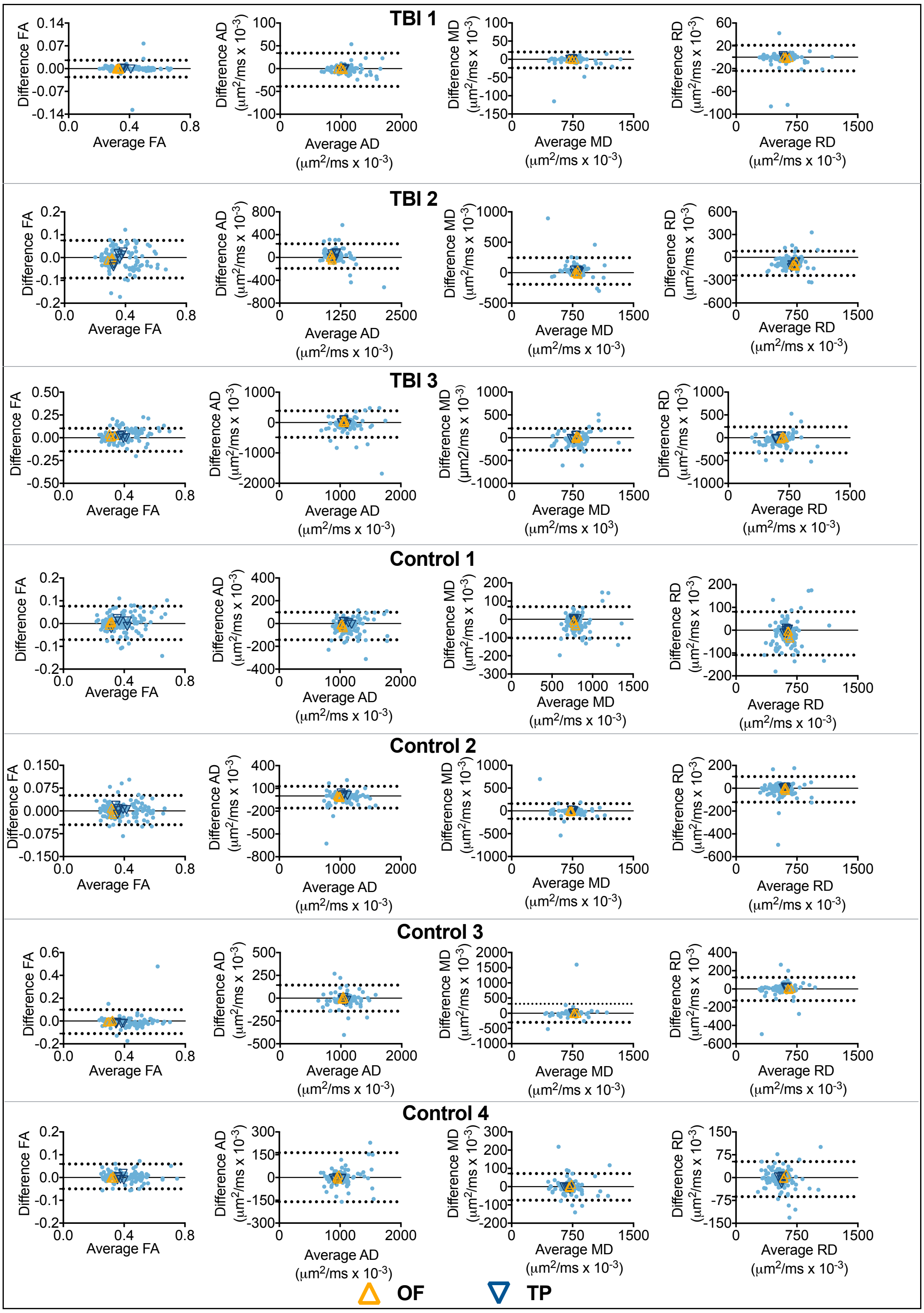

Fig. S6. Test-retest reliability of DTI with 1.25 mm isotropic voxel size in inferior brain white matter tracts. Bland-Altman (BA) plots showing overall robust agreement across all the diffusion metrics and study participants with all the values within the 95 % confidence interval with the exception of very few outliers. Each symbol represents a white matter region of interest from the DTI Studio parcellation. The orange and dark blue triangles in the BA plots show the agreement between scans in the OF and TP, respectively. The OF included the left and right lateral fronto-orbital gyrus [LFOG] and middle fronto-orbital gyrus [MFOG] DTI Studio parcellation

tracts, while the TP included the left and right superior temporal gyrus (STG), middle temporal gyrus [MTG], and inferior temporal gyrus [ITG]. Abbreviations: FA, fractions anisotropy; AD, axial diffusivity; MD, mean diffusivity; RD, radial diffusivity; OF, orbitofrontal; TP, temporal pole.

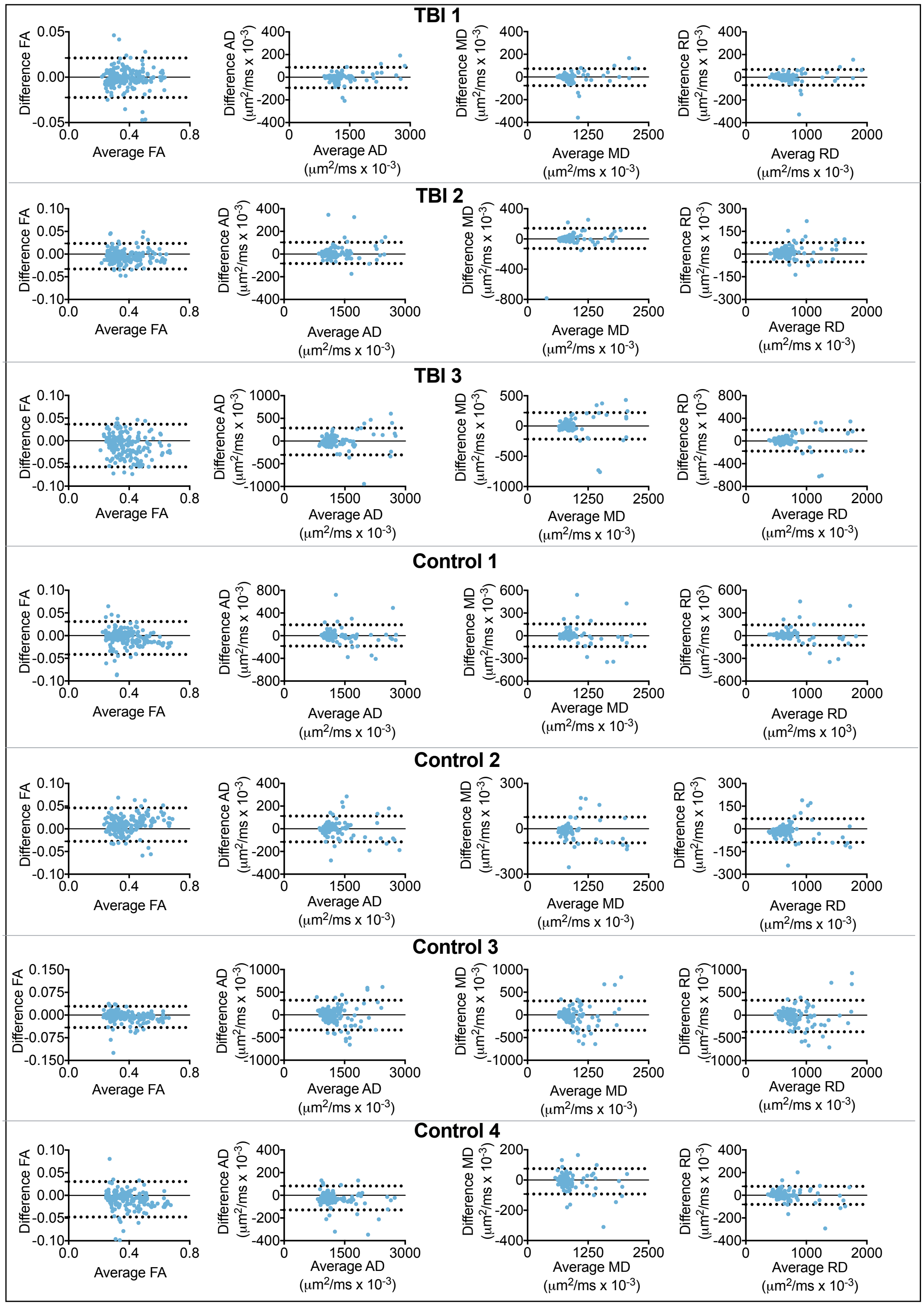

Fig. S7. Test-retest reliability of 1.75 mm isotropic voxel size DTI. Bland-Altman plots showing overall robust agreement across all the diffusion metrics and study participants with all the values within the 95 % confidence interval with the exception of very few outliers. Each symbol represents a white matter region of interest from the DTI Studio parcellation. Abbreviations: FA, fractional anisotropy; AD, axial diffusivity; MD, mean diffusivity; RD, radial diffusivity.

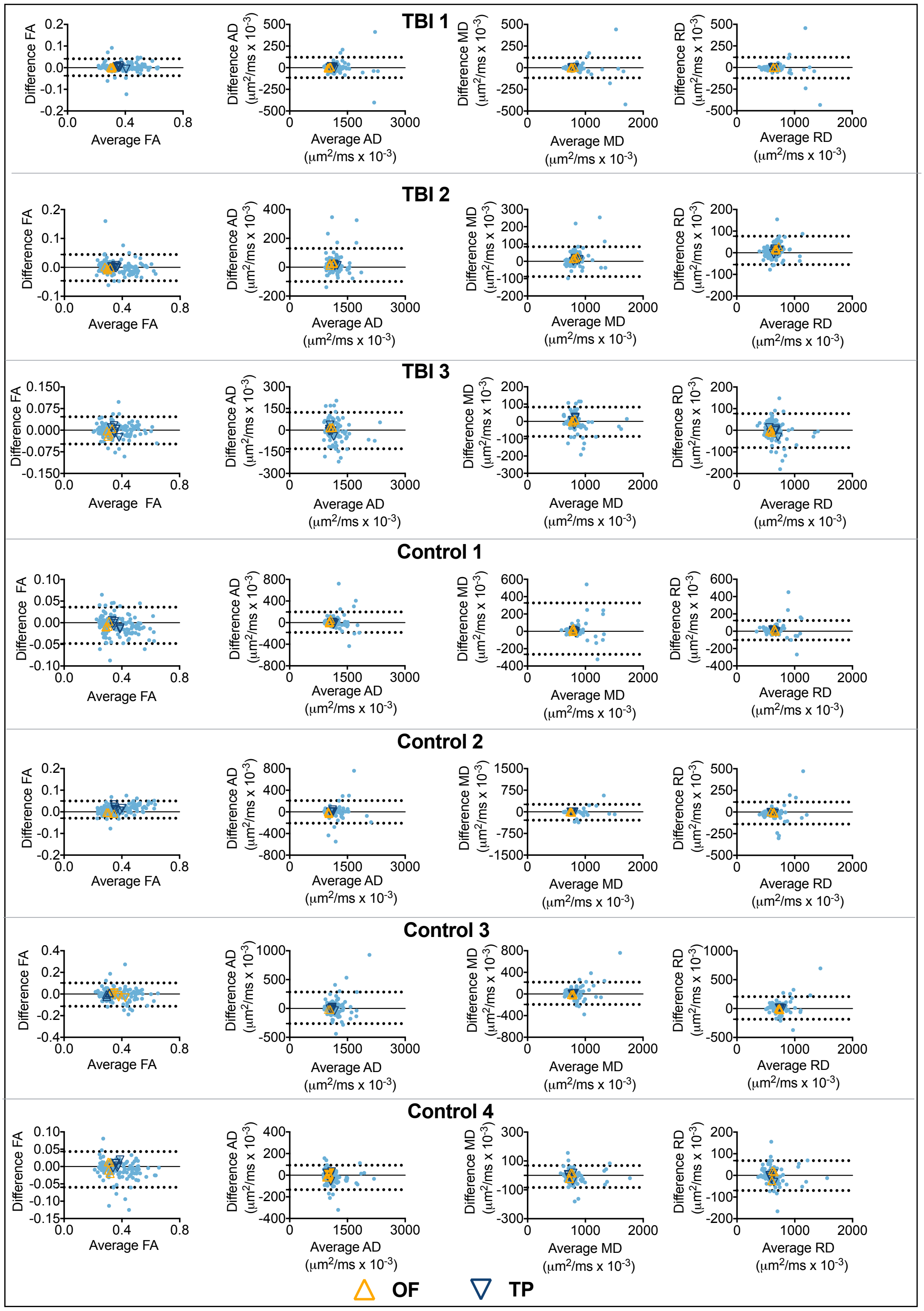

Fig. S8. Test-retest reliability of 1.75 mm isotropic voxel size DTI in inferior brain white matter tracts. Bland-Altman plots showing overall robust agreement across all the diffusion metrics and study participants with all the values within the 95 % confidence interval with the exception of very few outliers. Each symbol represents a white matter region of interest from the DTI Studio parcellation. The orange and dark blue triangles in the BA plots show the agreement between scans in the OF and TP, respectively. The OF included the left and right lateral fronto-orbital gryus [LFOG] and middle fronto-orbital gyrus [MFOG] DTI Studio parcellation tracts, while the TP

included the left and right superior temporal gyrus (STG), middle temporal gyrus [MTG], and inferior temporal gyrus [ITG]. Abbreviations: FA, fractions anisotropy; AD, axial diffusivity; MD, mean diffusivity; RD, radial diffusivity; OF, orbitofrontal cortex; TP, temporal pole.

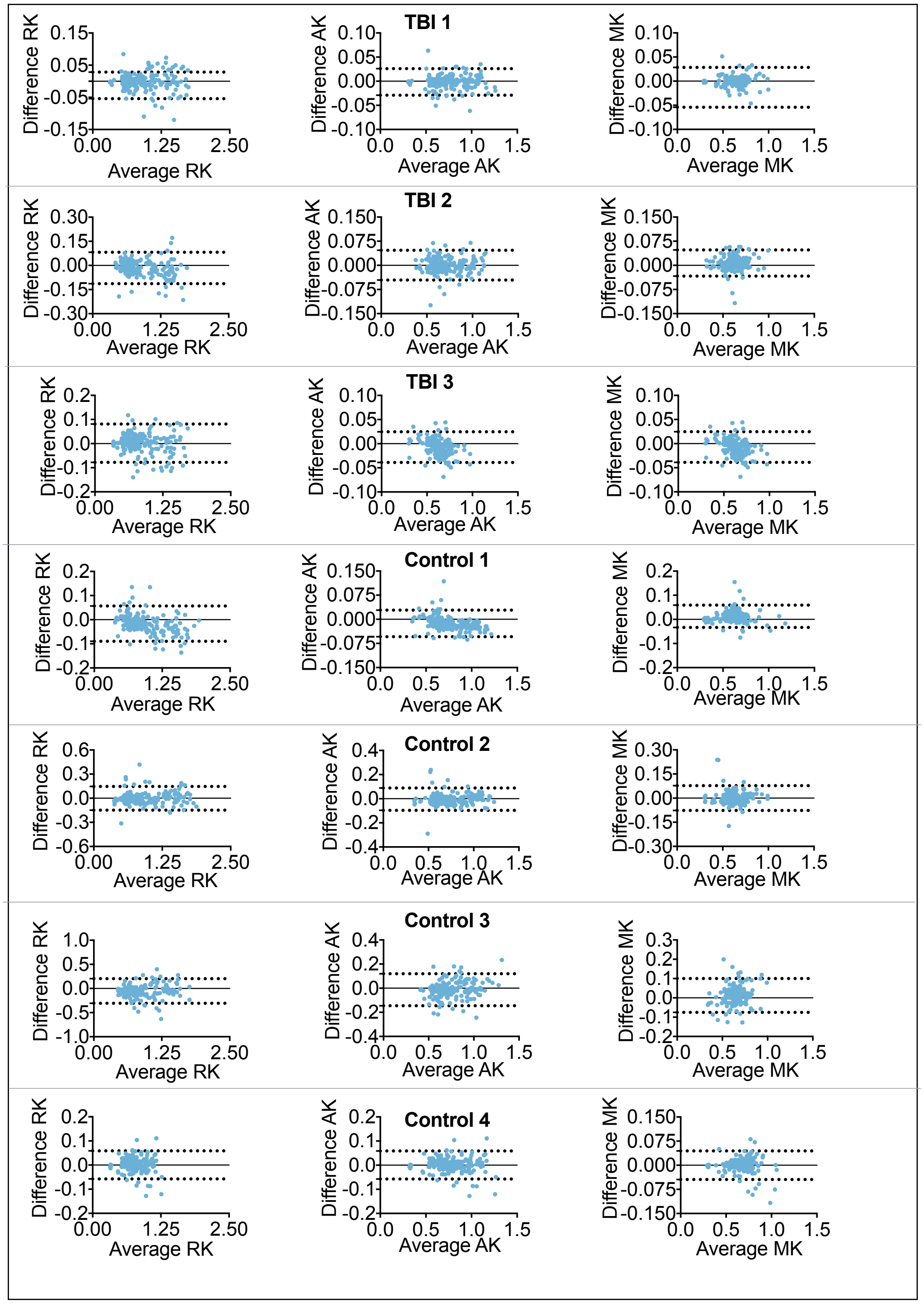

Fig. S9. Reliability of 1.75 mm isotropic voxel diffusion kurtosis imaging. Bland-Altman plots showing overall robust agreement across all the diffusion kurtosis metrics and study participants with all the values within the 95 % confidence interval with the exception of very few outliers. Each symbol represents a white matter region of interest from the DTI Studio parcellation. Abbreviations: AK, axial kurtosis; MK, mean kurtosis; RK, radial kurtosis.

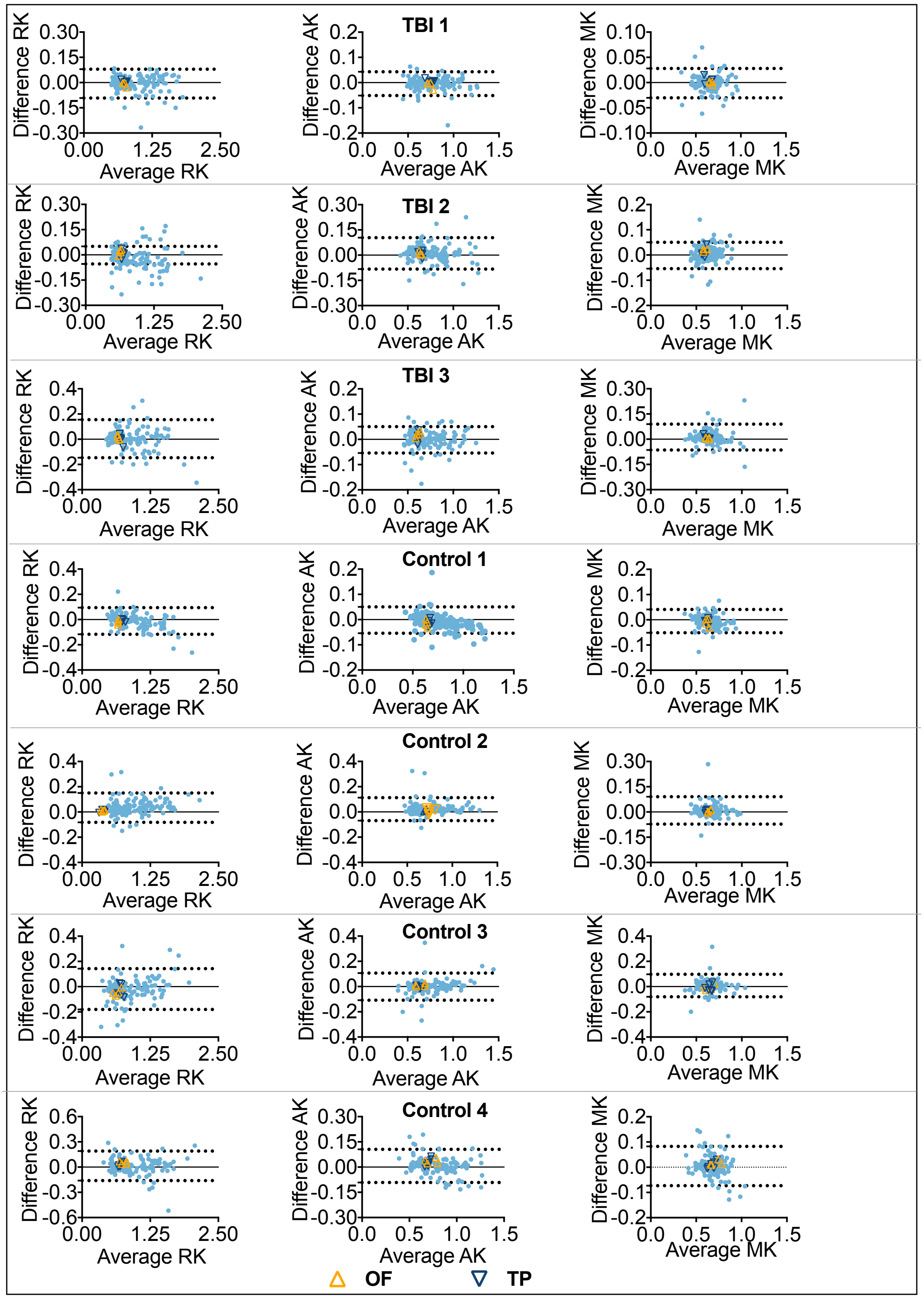

Fig. S10. Test-retest reliability of 1.75 mm isotropic voxel diffusion kurtosis imaging in inferior brain white matter tracts. Bland-Altman plots showing overall robust agreement across all the diffusion kurtosis metrics and study participants with all the values within the 95 % confidence interval with the exception of very few outliers. Each symbol represents a white matter region of interest from the DTI Studio parcellation. The orange and dark blue triangles in the BA plot show the agreement

between scans in the OF and TP, respectively. The OF included the left and right lateral fronto-orbital gryus [LFOG] and middle fronto-orbital gyrus [MFOG] DTI Studio parcellation tracts, while the TP included the left and right superior temporal gyrus (STG), middle temporal gyrus [MTG], and inferior temporal gyrus [ITG]. Abbreviations: AK, axial kurtosis; MK, mean kurtosis; RK, radial kurtosis; OF, orbitofrontal; TP, temporal pole.
